# One-Pot Synthesis of Disperse Dyes Under Microwave Irradiation: Dyebath Reuse in Dyeing of Polyester Fabrics

**DOI:** 10.3390/molecules17044266

**Published:** 2012-04-10

**Authors:** Alya M. Al-Etaibi, Morsy A. El-Apasery, Huda M. Mahmoud, Nouria A. Al-Awadi

**Affiliations:** 1 Natural Science Department, College of Health Science, Public Authority for Applied Education and Training, P.O. Box 14281, Fayha 72853, Kuwait; 2 Chemistry Department, Faculty of Science, Kuwait University, P.O. Box 5969, Safat 13060, Kuwait; 3 Dyeing, Printing and Textile Auxiliaries Department, Textile Research Division, National Research Centre, Dokki, Giza 12622, Egypt; 4 Department of Biological Sciences, Faculty of Science, Kuwait University, P.O. Box 5969, Safat 13060, Kuwait

**Keywords:** dyebath reuse, polyester fiber, disperse dyes, enaminones, microwave, X-ray crystallographic analysis

## Abstract

A series of 4-hydroxyphenylazopyrazolopyrimidine disperse dyes were prepared via one-pot reactions of *p*-hydroxyphenylhydrazone, hydrazine hydrate, and acetylacetone or enaminones using microwave irradiation as an energy source. Structural assignments of the dyes were confirmed by X-ray crystallographic structure determination. Instead of discharging the dyebath after each dyeing cycle, the residual dyebath was spectrophotometrically analyzed and then pH readjusted for a repeat dyeing with longer time. Fastness of the dyed samples was measured after each recycle. Most of the dyed fabrics tested displayed good light fastness and excellent fastness to washing and perspiration. Finally, the biological activity of the synthesized dyes against Gram positive bacteria, Gram negative bacteria and yeast were evaluated.

## 1. Introduction

The use of disperse dyes has been continuously increased in the textile industry since the discovery of synthetic fibers. These dyes can be applied to most synthetic fibres using simple exhaustion techniques. Disperse azo dyes, in particular, cause environmental concern due to their widespread use [1]. Reuse of water in textile processes has been a subject of research and development work in recent years [2,3,4,5]. The incentives for reuse of water are great, since there is a potential for reduction of both water requirements and wastewater treatment costs. The idea of dyebath renovation and reuse has started in the middle 1970s when energy costs became a critical factor in overall manufacturing costs. Dyebath renovation and reuse has been shown to be an effective method of cost reduction. One of the approaches to reuse the dye bath is to reconstitute the dye bath by adding the required amount of dyes and chemicals after analyzing the dye liquor. This method is applicable only if the dyeing process does not change the characters of the residual dye in the bath such as disperse dyes [6]. Dyebath reuse has long been recognized as a stratagem in pollution prevention and reduction of water, energy, and chemicals. The principle work on dyebath reuse has included the pilot and laboratory scales dyeing of polyester with disperse, in pilot scale experiments, dyebaths have been reused over 30 times [7,8,9,10,11]. In the present study, after the original dyeing process, polyester fibres was dyed with disperse dyes in a dyebath reuse system using microwave irradiation [12,13,14,15]. The objective of the study was to save water and chemicals and to reduce the quantities of effluent discharged during the dyeing of polyester fibers. Instead of discharging the dyebath after each dyeing cycle, the residual dyebath was spectrophotometrically analyzed and then pH readjusted for a repeated dyeing with longer time. Also, the present study was undertaken to investigate the biological activity of the synthesized dyes against *Escherichia coli *and *Serratia* sp. (Gram negative bacteria), *Bacillus subtilus* and *Staphylococcus auerus* (Gram positive bacteria) and *Candida albicans *and *Saccharomyces cerevisiae* (Yeast).

## 2. Results and Discussion

### 2.1. Synthesis

Recently we have reported the synthesis of new disperse dyes [16] which showed significant results in dyeing of polyester fibres. Herein, in an attempt to improve and facilitate the synthesis of these dyes, we report a new strategy for the preparation of these disperse dyes by using one-step reaction of *p*-hydroxyphenylhydrazone **4**, hydrazine hydrate, and acetylacetone or enaminones **8a**,**b**.

**Scheme 1:**
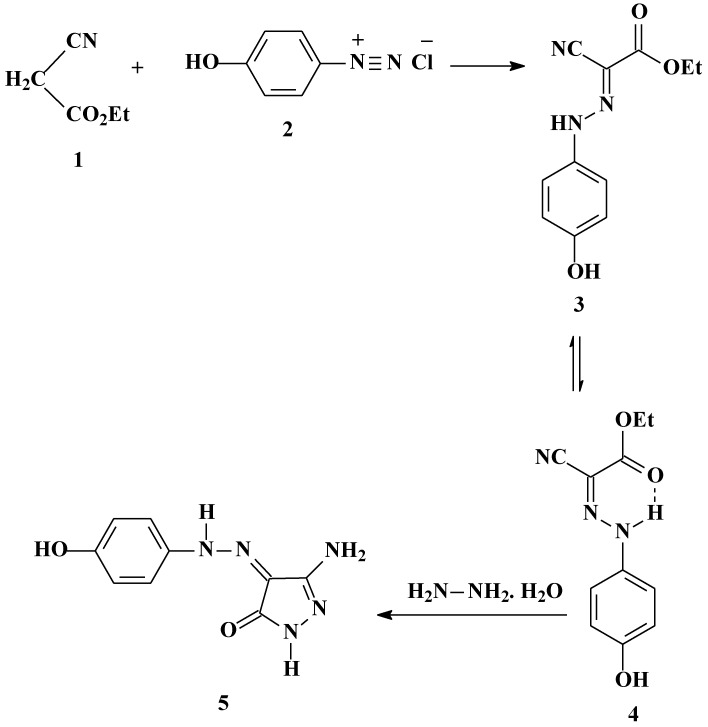
Preparation of 5-Amino-4-[(4-hydroxyphenyl)hydrazono]-2,4-dihydropyrazol-3-one (**5**).

**Figure 1:**
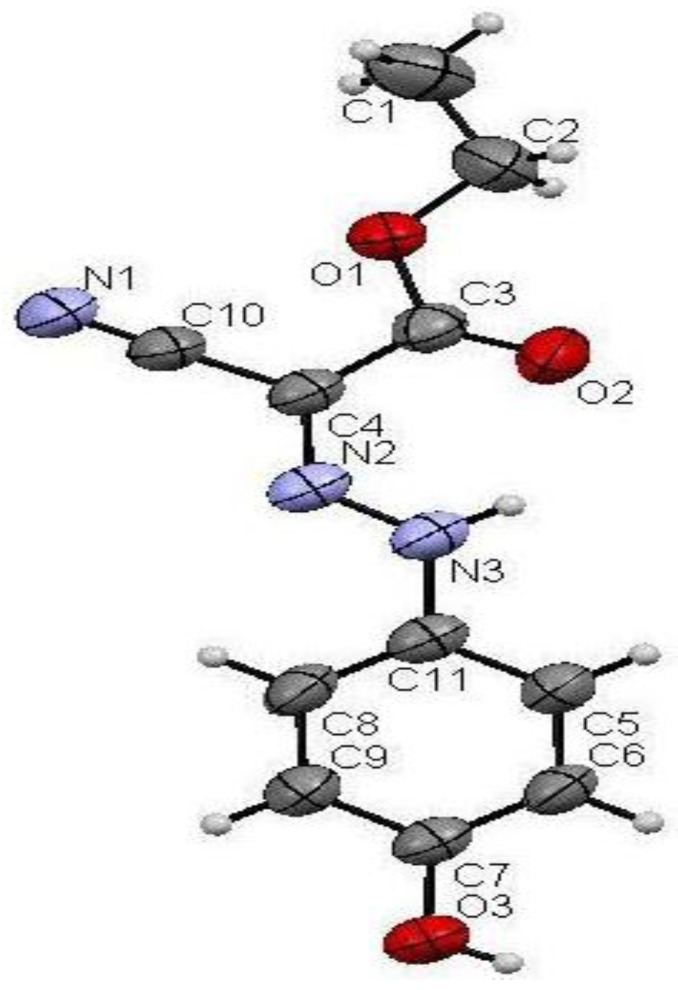
Ortep plot of the X-ray crystallographic structure of **4**.

*p*-Hydroxyphenylhydrazone **4** was formed by addition under mild conditions of ethyl cyanoacetate (**1**) to 4-hydroxybenzenediazonium chloride (**2**), in ethanolic sodium acetate solution, that readily affords the corresponding *p*-hydroxyphenylhydrazone **4**. The existence of this hydrazone in the isomeric form **3** was ruled out based on X-ray crystallographic structure determination. (*cf.* Scheme 1 and Figure 1) [17]. *p*-Hydroxyphenylhydrazone **4** undergoes a smooth reaction with hydrazine hydrate by heating in a focused microwave oven at 120 °C for 5 min to yield 5-amino-4-[(4-hydroxyphenyl)-hydrazono]-2,4-dihydropyrazol-3-one (**5**).

**Scheme 2:**
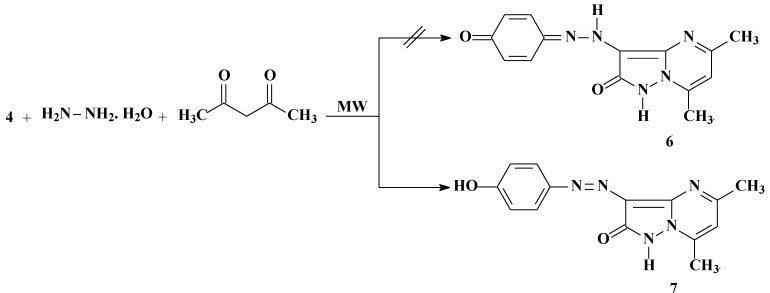
Synthesis of 3-(4-Hydroxyphenylazo)-5,7-dimethylpyrazolo[1,5-a]pyrimidin-2-one(**7**).

**Figure 2:**
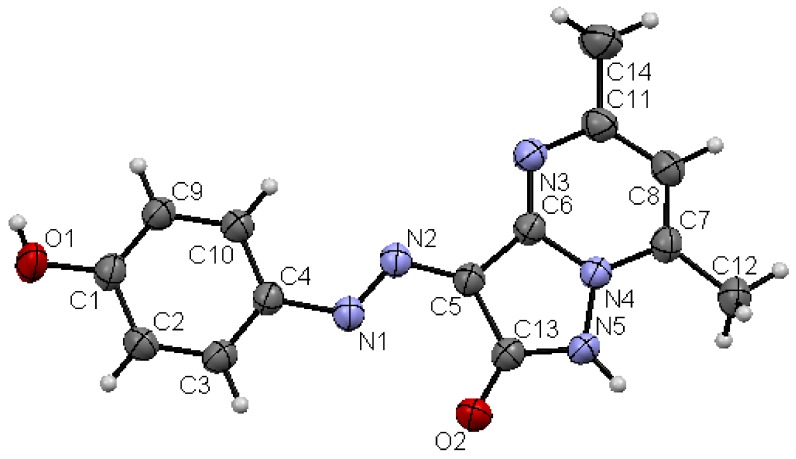
Ortep plot of the X-ray crystallographic structure of **7**.

We have previously reported that **5** reacts with acetylacetone to yield the pyrazolo[1,5-a]pyrimidine **7** [16]. We now report one-pot synthesis of **7** in better yield from *p*-hydroxyphenylhydrazone **4**, hydrazine hydrate, and acetylacetone. Compound **7** has the potential of existing in tautomeric form **6**. The existence of this disperse dye in the tautomeric form **6** was ruled out based on X-ray crystallographic structure determination. (*cf.* Scheme 2 and Figure 2) [17]. 

**Scheme 3:**
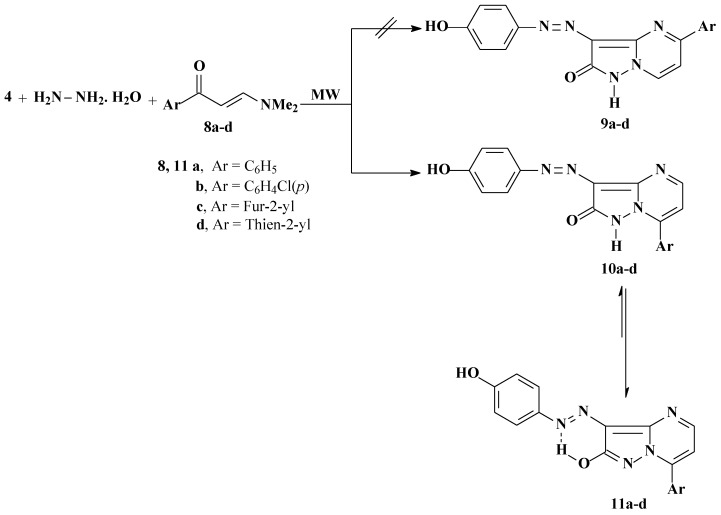
Synthesis of pyrazolo[1,5-a]pyrimidin-2-ol derivatives (**11a**–**d**).

**Figure 3:**
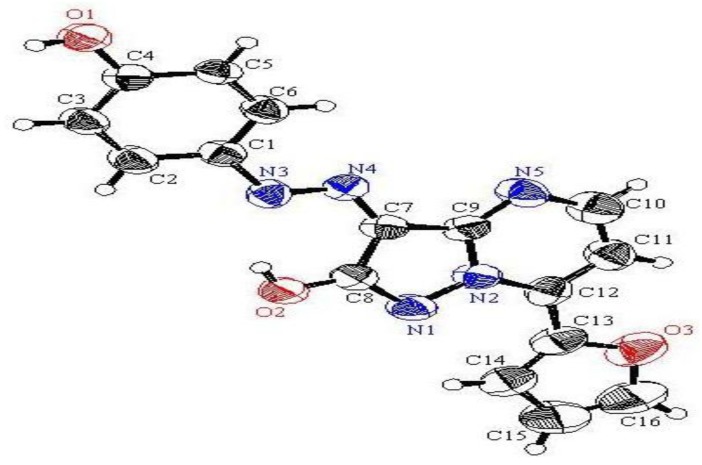
Ortep plot of the X-ray crystallographic structure of **11c**.

We have also previously reported that **5** reacts with enaminones **8a**–**d** to yield the pyrazolo[1,5-a]pyrimidine derivatives **10a**–**d** [16]. We now report that **10a**–**d** could be directly formed by one-pot reactions of hydrazone **4**, hydrazine hydrate, and enaminones **8a**–**d** in a focused microwave oven at 130 °C for 5 min. The possible formation of regioisomers **9** in the reactions of hydrazone **4**, hydrazine hydrate with enaminones **8a**–**d** is ruled out based on X-ray crystallographic structure determination (Figure 3). Although these disperse dyes are believed to exist mainly in the keto-form **10a**–**d**, the predominance of **11a**–**d** (as indicated by the X-ray crystallographic structure determination) is attributed to stabilization of the products by hydrogen bonding. The fact that compound **7** prefers the keto-form, while compounds **11a**–**d** prefer the enol-form may be due to the replacement of a methyl group in compound **7** by aromatic and heteroaromatic substituents with extended conjugation in the enol-form (*cf.* Scheme 3) [17].

### 2.2. Dyeing

The functionalized disperse dyes **5**, **7** and **11a**–**d** were applied to polyester fabrics at 1–6% shade by a high-temperature pressure technique at 130 °C by employing focused microwave irradiation as an energy source, and a range of color shades was obtained, varying from yellowish-orange to dark orange. The dyeing on the polyester fabrics were evaluated in terms of their fastness properties (e.g., fastnesses to washing, perspiration, and light). The physical data for the dyed fibers, given in Table 1, Table 2, Table 3, reflect the efficiency of the microwave irradiation, leading to a large increase in dye uptake and dyeing rate along with a performance of dye leveling and color homogeneity as compared to conventional methods.

**Table 1:** Color strengths of azo disperse dyes on polyester fabrics of first dyeing.

Dye No	Color shade on polyester	Color strength (K/S) % Dye o.m.f.					
		1	2	3	4	5	6
**5**	Yellowish-orange	1.17	2.12	2.47	2.49	4.25	4.66
**7**	Pale orange	2.19	3.79	4.09	6.71	6.91	6.96
**11a**	Dark orange	5.66	5.95	7.12	8.31	10.30	10.57
**11b**	Dark orange	5.43	5.81	7.91	8.48	10.04	12.89
**11c**	Orange	4.40	4.64	4.85	6.01	6.25	6.56
**11d**	Orange	4.08	4.73	6.02	7.06	7.47	8.85

**Table 2:** Color strengths of azo disperse dyes on polyester fabrics of reused dyebaths.

Dye No	Color shade on polyester	Color strength (K/S) % Dye o.m.f.					
		1	2	3	4	5	6
**5**	Yellowish-orange	1.02	1.67	2.42	2.09	3.28	3.99
**7**	Pale orange	2.12	3.56	4.08	5.70	5.77	6.79
**11a**	Dark orange	4.76	5.47	5.56	6.20	6.89	8.78
**11b**	Dark orange	3.06	3.99	4.30	4.76	6.66	8.59
**11c**	Orange	3.45	3.54	3.48	4.35	4.55	5.41
**11d**	Orange	2.59	3.95	4.17	5.63	6.44	8.28

**Table 3:** Fastness properties of monoazo disperse dyes on polyester fabrics.

Dye	Dye o.m.f. %	Wash fastness			Perspiration fastness						Light fastness
					Alkaline			Acidic			
		Alt	SC	SW	Alt	SC	SW	Alt	SC	SW	
**5**	**1%**	5	5	5	5	5	5	5	5	5	3–4
**7**		5	5	5	5	5	5	5	5	5	2
**11a**		5	5	5	5	5	5	5	5	5	3
**11b**		5	5	5	5	5	5	5	5	5	2–3
**11c**		4–5	4	4–5	5	5	4–5	5	5	5	2–3
**11d**		4–5	4–5	4–5	5	5	5	5	5	5	2–3
**5**	**2%**	5	5	5	5	5	5	5	4	5	4
**7**		5	5	5	5	5	5	5	5	5	2
**11a**		5	5	5	5	5	5	5	5	5	2–3
**11b**		5	5	5	5	5	5	5	4	5	3
**11c**		4–5	4	4–5	5	5	5	4–5	4–5	4–5	2–3
**11d**		4–5	4–5	4–5	5	5	5	5	5	5	2–3
**5**	**3%**	5	5	5	5	5	5	5	5	5	3–4
**7**		5	5	5	5	5	5	5	5	5	2–3
**11a**		4–5	4–5	4–5	5	5	5	5	4–5	5	2–3
**11b**		5	4–5	4–5	5	5	5	5	5	5	3
**11c**		4–5	4–5	4–5	5	5	5	5	5	5	2–3
**11d**		4–5	4	4–5	5	5	5	5	5	5	2–3
**5**	**4%**	5	5	5	5	5	5	5	5	5	4
**7**		5	4	4	5	5	5	5	5	5	2–3
**11a**		5	5	5	5	5	5	5	5	5	2–3
**11b**		5	5	5	5	5	5	5	5	5	3–4
**11c**		5	4–5	4–5	5	4–5	5	5	5	5	2–3
**11d**		5	4–5	4–5	4–5	4–5	5	4–5	4–5	5	2
**5**	**5%**	5	5	5	5	5	5	5	5	5	4–5
**7**		5	5	5	5	5	5	5	5	5	2–3
**11a**		5	4–5	4–5	5	5	5	5	5	5	3
**11b**		5	5	5	5	5	5	5	5	5	2–3
**11c**		4–5	4–5	4–5	5	5	5	5	5	5	2–3
**11d**		5	4	4	5	5	4–5	5	5	5	3
**5**	**6%**	5	5	5	5	5	5	5	5	5	4
**7**		5	5	5	5	5	5	5	5	5	2–3
**11a**		4–5	4–5	4–5	5	4–5	5	5	4–5	5	2–3
**11b**		4–5	4–5	4–5	5	4–5	5	5	5	5	3–4
**11c**		4–5	4–5	4–5	5	5	5	5	5	5	3
**11d**		4–5	4	4–5	5	5	4–5	5	5	5	3

Alt = alteration; SC = staining on cotton; SW = staining on wool.

#### 2.2.1. Color Strength

Most of the auxiliary chemicals used in dyeing of polyester are generally non-reactive and are not absorbed by the substrate being dyed, so they are not consumed or removed during the dyeing cycle [18,19,20,21]. Reuse of dyebaths generally depends on addition of the amount of dye lost each time. In this study we planned to reuse the dyebath without any addition of the used dye by increasing the time of dyeing. The data in Table 1 showed K/S of first dyeing for 60 min at 130 °C in a focused microwave oven, while that in Table 2 showed K/S of reused dyebath for 90 min at 130 °C in a focused microwave oven. The physical data and the overall color strength of dyed fabrics between the first and reused dyeing are shown in Table 1, Table 2, Table 3. After reuse of the dyebath, color consistency of dyeings remained acceptable, which are hard to distinguish with human eyes. In particular, Table 1 and Table 2 clearly show that the magnitude of color strength obtained using dye **11d** is much larger than that for **11c**. 

#### 2.2.2. Color Fastness

Fastness data, obtained by measuring color intensity changes in the dyed polyester fabrics of reused dyebath, are given in Table 3. It shows the results of color fastness to washing, light, and perspiration, the rating for color fastness to light and washing were good to excellent, compared to that of the first dyeing of our previous published work [16]. The rating for color fastness to perspiration was excellent. These results clearly show that no deterioration of the color fastness occurs over the series of reused dyebaths. In addition the results obtained showed that dyed fabrics have good fastness properties may be due to: (**a**) the absence of solubilizing groups, which affects solubility, and wash ability of the dye-out of the fabrics; (**b**) the size of the dye molecule is considered relatively big; (**c**) the good intra-fiber diffusion of the dye molecules inside the fabrics.

### 2.3. Antimicrobial Activities

The antimicrobial activities of the synthesized dyes were screened against selected bacteria and fungi by the agar well diffusion method and their inhibition zones diameters, given in Table 4, reveal that all of the tested dyes showed positive antimicrobial activities against at least one of the tested microorganisms. Two of the compounds, **4** and dye **5**, showed strong activities, with significant inhibition zones >10 mm, against Gram positive bacteria, while others showed moderate to weak activities against the tested microorganisms. It worth noting that all tested compounds inhibited the growth of the *Candidia albicans*.

**Table 4:** Diameter of the zones of inhibition of the tested compoundsthat showed weak to strong antimicrobial against microorganisms.

Compound number	Inhibition zone diameter (Nearest mm)					
	prokaryotic organisms				eukaryotic organisms	
	*B. subtilus*	*S. aureus*	*E. coli*	*Serratia sp.*	*C. albicans*	*S. cerevisiae*
	Mean ± SD	Mean ± SD	Mean ± SD	Mean ± SD	Mean ± SD	Mean ± SD
Strong antimicrobial						
**4**	11(0.1)	13(0.3)	NI	4 (0.1)	8(0.3)	1(0.3)
**5**	11(0.2)	12(0.1)	NI	NI	1(0.2)	NI
						
Moderate antimicrobial						
**11a**	7(0.2)	7(0.2)	NI	NI	1(0.1)	NI
						
Weak antimicrobial						
**7**	4(0.2)	NI	NI	NI	4(0.1)	NI
**11b**	2(0)	NI	NI	NI	2(0.1)	NI
**11c**	2(0)	NI	NI	2(0.1)	3(0.2)	NI
**11d**	2(0)	3(0.1)	NI	NI	3(0.2)	1(0.1)
**Ampicillin** *	7	30	15	26		
**Cyloheximide** **					NI	30

(NI) no inhibition, * Ampicillin: Antibacterial (100 mg·mL^−1^); ** Cycloheximide: Antifungal (100 mg·mL^−1^), SD = Standard Deviation.

**Figure 4:**
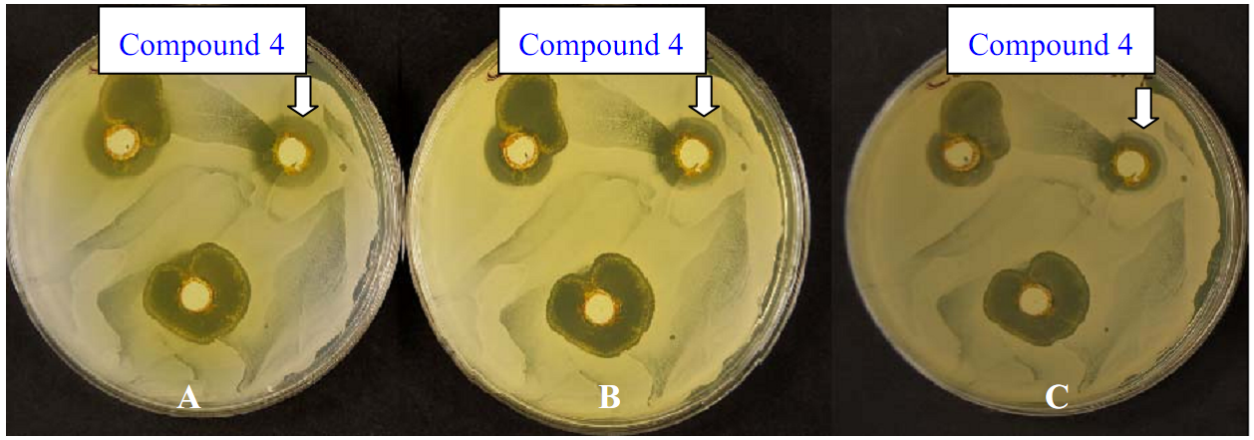
*Candida albicans* treated with 10 mg·mL^−1^ of compound **4** after one day “A”, three day “B” and six days “C” of incubation.

**Figure 5:**
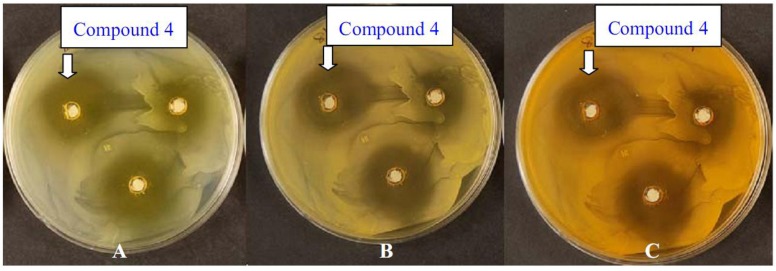
*Staphylococcus aureus* treated with 10 mg·mL^−1^ of compound **4** after one day “A”, three day “B” and six days “C” of incubation.

In addition, Figure 4 shows that *Candida albicans* re-grew in the formed zone surrounding the wells contain the compound **4**. This may reflect a cytostatic effect of the chemicals rather than cytolytic effects. The same effect was also noted for the other tested microbes, for instance in Figure 5 the zone surrounding the wells of the plates inoculated with *Staphylococcus aureus* showed also cytostatic effect for the starting chemical. Note that the plate color changed with the increase in the incubation time, indicating a complete diffusion of the chemical used in the agar as the incubation period increased.

The cytolytic effects of dyes number **5**, **7**, and **11a** on *Candida albicans*, where after one, three and six days of incubation, the inhibition zone did not change is worth noting, while the cytostatic effect of the same dyes is clear on the plates inoculated with *Bacillus subtilus. *The growth of *B. subtilus* resumed in the inhibited area after six days of incubation which may indicate that as the concentration/toxicity of dyes number **5**, **7**, and **11a** was reduced due to possible evaporation of these dyes or diffusion in the media, the effect of these dyes on *B. subtilus* decreased and the organisms started growing again. The results obtained for the synthesized compounds in this study support the findings of the other researchers who showed that pyrazole and pyrazolo[1,5-a]pyrimidine cores have various biological activities. Pyrazolo[1,5-a]pyrimidines were proved by many researchers to possess biological effects, including antimicrobial activities (herbicidal and fungicidal activities) [22]. Therefore, the new synthesized classes showed promising results for possessing the potentials to be utilized for medicinal purposes. Currently, we are inspecting the biological activity of the dyed polyester with pyrazolopyrimidines disperse dyes against Gram positive bacteria, Gram negative bacteria and yeast.

## 3. Experimental

### 3.1. General

All melting points were recorded on a Gallenkamp apparatus and are uncorrected. IR spectra were recorded in KBr disks on a Perkin Elmer System 2000 FT-IR spectrophotometer. ^1^H- and ^13^C-NMR spectra were recorded on a Bruker DPX 400 MHz super-conducting NMR spectrometer. Mass spectra were measured on a VG Auto-spec-Q instrument (high resolution, high performance, tri-sector GC/MS/MS) and by LC-MS using an Agilent 1100 series LC/MSD with API-ES/APCI ionization mode. Microanalyses were performed on a LECO CH NS-932 Elemental Analyzer. The microwave oven used is a single mode cavity Explorer Microwave (CEM Corporation, Matthews, NC, USA) and irradiate in heavy-walled Pyrex tube (capacity 10 mL and 80 mL for dyeing). The color strengths (K/S) of the dyed polyester fabrics and the color fastness to light were evaluated at the Dyeing, Printing and Textile Auxiliaries Department, Textile Research Division, National Research Centre, Giza, Egypt. X-ray crystals were measured by single crystal X-ray crystallography-Rigaku Rapid II). Copies of original data can be provided by the authors upon request.

### 3.2. General Procedure for the Synthesis of Azo Disperse Dyes

*Cyano-[(4-hydroxyphenyl)hydrazono]-acetic acid ethyl ester *(**4**). A cold solution of the diazonium salt [10 mmol; prepared by adding a cold solution of sodium nitrite (0.7 g) in water (5 mL) to a solution of the p-aminophenol (1.09 g, 10 mmol) in conc. HCl (5 mL)] was added to a cold solution of ethyl cyanoacetate (1.0 g, 10 mmol) in EtOH (10 mL) containing NaOAc (3 g). The mixture was stirred at room temperature for 1 h, and the solid precipitate that formed was collected by filtration and crystallized from EtOH to give brown crystals, yield (87%), m.p. 274 °C; MS: *m/z* = 233 (M^+^, 100%), 207 (5%), 159 (40%), 121 (12%), 108 (100%),.81 (25%), 65 (14%); IR: 3420 (OH), 3314 (NH), 2234 (CN), 1684 (CO) cm^–1^; ^1^H-NMR (CDCl_3_) δ = 1.40 (t, 3H, *J* = 7.2 Hz, CH_3_). 4.36 (q, 2H, *J* = 7.2 Hz, CH_2_), 5.02 (s, 1H, OH), 6.87–6.91 (m, 2H, arom-H), 7.24–7.28 (m, 2H, arom-H), 13.22 (s, 1H, NH).

*5-Amino-4-[(4-hydroxyphenyl)hydrazono]-2*,*4-dihydropyrazol-3-one* (**5**). A mixture of **3** (2.33 g, 10 mmol) and hydrazine hydrate (1.5 mL) in ethanol (2 mol) was irradiated in a microwave oven at 120 °C for 5 min. The solid formed was collected and crystallized from ethanol to give reddish-brown crystals, yield (74%), m.p. 263 °C (lit.[16] m.p. 263 °C).

*General procedure for the synthesis of pyrazolo[1,5-a]pyrimidines* **7** and **11a**–**d**. A mixture of **4** (2.33 g, 10 mmol), hydrazine hydrate (1.5 mL) and ethanol (2 mL) were irradiated in a microwave oven at 130 °C for 3 min. Acetylacetone, or enaminones **8a**–**d** (1 mmol) were then added and the heating continued at 130 °C for additional 2 min. The mixture was then poured into ice water. The solid formed was collected and crystallized from the appropriate solvent (see below).

*3-(4-Hydroxyphenylazo)-5*,*7-dimethylpyrazolo[1,5-a]pyrimidin-2-one *(**7**). Red crystals from DMF, yield (88%), m.p. 277–278 °C (lit.[16] m.p. 277–278 °C). 

*3-(4-Hydroxyphenylazo)-7-phenylpyrazolo[1,5-a]pyrimidin-2-ol *(**11a**). Red crystals from DMF, yield (76%); m.p. 273–274 °C; MS: *m/z* = 331 (M^+^, 100%), 238 (50%), 182 (20%); IR: 3432 (OH) cm^–1^; ^1^H-NMR (DMSO-*d_6_*) δ = 3.36 ppm (br, 1H, OH, D_2_O exchangeable), 6.90 (d, 2H, *J *= 7.2), 7.38 (d, 1H, *J *= 4.2 Hz), 7.64 (m, 3H), 7.71 (d, 2H, *J *= 7.2 Hz), 8.07 (d, 2H, *J* = 7.2 Hz), 8.70 (d, 1H, *J* = 4.2 Hz), 10.02 (s, 1H, OH, D_2_O exchangeable).

*7-(4-Chlorophenyl)-3-(4-hydroxyphenylazo)pyrazolo[1,5-a]pyrimidin-2-ol* (**11b**). Red crystals from DMF, yield (85%); m.p. 285–286 °C; MS: *m/z* = 365 (M^+^, 100%), 336 (6%), 272 (48%), 244 (12%), 216 (22%), 180 (7%), 149 (22%), 93 (8%); IR: 3423 (OH) cm^–1^; ^1^H-NMR (DMSO-d_6_) δ = 3.38 ppm (br, 1H, OH, D_2_O exchangeable), 6.88 (d, 2H, *J* = 8.0 Hz), 7.40 (d, 1H, *J* = 3.3 Hz), 7.70 (d, 4H,*J* = 8.0 Hz), 8.13 (d, 2H, *J* = 8.4 Hz), 8.69 (d, 1H, *J* = 3.2 Hz), 10.03 (s, 1H, OH, D_2_O exchangeable); ^13^C-NMR (DMSO-*d_6_*) δ = 161.2, 158.8, 151.4, 145.0, 144.5, 143.4, 136.0, 131.5, 128.8, 128.6, 122.4, 115.8, 114.2, 110.4. 

*7-Furan-2-yl-3-(4-hydroxyphenylazo)pyrazolo[1,5-a]pyrimidin-2-ol* (**11c**). Red crystals from DMF, yield (83%); m.p. 291–292 °C; MS: *m/z* = 321 (M^+^, 100%), 228 (36%), 200 (12%), 172 (13%), 116 (6%), 93 (7%); IR: 3435 (OH) cm^–1^;^1^H NMR (DMSO-*d_6_*) δ = 3.84 (br, 1H, OH, D_2_O exchangeable), 6.89 (d, 2H, *J *= 8.4 Hz), 6.95 (s, 1H), 7.59 (d, 1H, *J *= 4.4 Hz), 7.68 (d, 2H, *J* = 8.4 Hz), 8.14 (d, 1H, *J* = 3.2 Hz), 8.22 (s, 1H), 8.63 (d, 1H, *J* = 4.8 Hz), 10.00 (s, 1H, OH, D_2_O exchangeable); ^13^C-NMR (DMSO-*d_6_*) δ =162.0, 159.0, 150.7, 148.3, 145.5, 143.4, 141.8, 134.7, 122.4, 121.2, 116.3, 115.2, 114.7, 114.0, 105.9.

*3-(4-Hydroxyphenylazo)-7-thiophen-2-yl-pyrazolo[1,5-a]pyrimidin-2-ol *(**11d**). Red crystals from DMF, yield (76%); m.p. 284–285 °C; MS: *m/z* = 337 (M^+^, 100%), 308 (5%), 244 (40%), 218 (14%), 187 (14%), 121 (13%), 65 (5); IR: 3444 (OH) cm^–1^; ^1^H-NMR (DMSO-*d_6_*) δ = 3.49 ppm (s, 1H, OH, D_2_O exchangeable), 6.89 (d, 2H, *J *= 8.8 Hz), 7.40 (t, 1H, *J* = 4.0 Hz), 7.66 (d, 2H, *J* = 8.4 Hz), 7.96 (d, 1H, *J* = 4.8 Hz), 8.19 (d, 1H, *J* = 4.4 Hz), 8.55 (d, 1H, *J *= 2.8 Hz), 8.61 (d, 1H, *J *= 4.8 Hz), 9.99 (s, 1H, OH, D_2_O exchangeable); ^13^C-NMR (DMSO-*d_6_*) 162.6, 158.7, 150.4, 146.0, 140.4, 136.7, 133.6, 133.5, 129.9, 128.3, 121.7, 116.4, 116.2, 108.3; Anal. Calcd. for C_16_H_11_N_5_O_2_S (337.4): C 56.96; H 3.29, N 20.76; S 9.50. Found: C 56.88; H 3.30; N 20.71; S 9.46.

### 3.3. High Temperature Dyeing Method *(HT)*

#### 3.3.1. Materials

Scoured and bleached polyester 100% (150 130 g/m^2^, 70/2 denier) was obtained from El-Shourbagy Co., Egypt. The fabric was treated before dyeing with a solution containing non-ionic detergent (Hostapal CV, Clariant-Egypt, 5 g/L) and sodium carbonate (2 g/L) in a ratio of 50:1 at 60 °C for 30 min, then thoroughly washed with water and air dried at room temperature.

#### 3.3.2. Dyeing

Dyeing of polyester fabrics was carried out at 130 °C for 60 min, under pressure in a focused microwave oven in a 20:1 liquor ratio and pH 5.5 in the presence of a 1:1 ratio of the dispersing agent sodium lignin sulphonate and the with a 1–6% shade. After dyeing, the fabrics were thoroughly washed and then subjected to a surface reduction cleaning [(3 g NaOH + 2 g sodium hydrosulphite)/L]. The samples were heated in this solution for 10 min. at 60 °C and then thoroughly washed and air-dried. Dyebath exhaustion (%) was determined using a UV/Visible-spectrophotometer.

#### 3.3.3. Dyebath Reuse Procedure

After dyeing, the dyebath was analyzed and reconstituted with the necessary amount of fresh water to maintain a constant liquor ratio of the original volume. Residual dyebath pH was measured in order to keep pH at 5.5. Second The dyeing was being carried out by raising the dye bath temperature from 20 to 130 °C at a rate of 30 °C/min and holding at this temperature for 90 min (in order to increase the dye uptake) before rapidly cooled to 50 at 10 °C/min. The dyed fabrics was then rinsed with cold water, reduction-cleared using sodium hydroxide (3 g/L) and sodium hydrosulphite (2 g/L) and soaped with 2% nonionic detergent (pH 8) at 50°C for 15 minutes to improve washing fastness.

### 3.4. Color Measurements and Analyses

#### 3.4.1. Color Measurements of the Dyed Fabrics

The color yields of the dyed samples were determined by using the light reflectance technique performed on a Perkin-Elmer (Lambda 3B) UV/VIS Spectrophotometer. The color strengths, expressed as K/S values, were determined by applying the Kubelka-Mink equation as follows:

                     K/S = [(1 − R)^2^ / 2R] − [(1 − R_o_)^2^ / 2R_o_]

where *R* = decimal fraction of the reflectance of the dyed fabric; *R_o_* = decimal fraction of the reflectance of the undyed fabric; *K* = absorption coefficient; *S* = scattering coefficient.

#### 3.4.2. Fastness Testing

The dyed fabrics were tested, employing ISO standard methods [23]. Wash fastness tests were carried out in accordance with ISO 105-C04 (1989), in which 5 g/L soap and 2 g/L soda ash solution were used at 95 °C for 30 min in the presence of 10 steel balls (liquor ratio 50:1) and color fastness to light (carbon arc), ISO 105-B02 (1988).

### 3.5. Antimicrobial Activities Test

The antimicrobial activities of seven different compounds were tested against six different microbial cultures using the agar-well diffusion technique [24]. Pure cultures of *Escherichia coli *and *Serratia* sp. (Gram negative bacteria), *Bacillus subtilus* and *Staphylococcus auerus* (Gram positive bacteria) and *Candida albicans *and *Saccharomyces cerevisiae* (Yeast) were employed in the test. An aliquot of each bacterial strain (0.1 mL) was inoculated and spread on nutrient agar (NA) while the yeast (0.1 mL) was spread on potato dextrose agar (PDA). The inoculated plates were supplied with 100 µL of each of the tested chemicals with a total final concentration of 10 mg·mL^−1^. The compounds were placed in 4 mm wells produced by sterile cork borer. The NA plates were incubated at 37 °C for 24 h while PDA plates were incubated at 25 °C for 24–48 h. The zones of inhibition around the wells were determined and the averages based on three replicas were recorded. Cycloheximide and ampicillin both used as references in the experiment, where cycloheximide known to inhibit eukaryotic organisms while ampicillin inhibits prokaryotes.

All plates were kept for six days after inoculation and the changes in the inhibition zone was monitored and documented by photography in order to determine on the cytolytic and cytostatic effect of the tested chemicals.

## 4. Conclusions

In summary, a series of 4-hydroxyphenylazopyrazolopyrimidine disperse dyes were synthesized via one-pot synthesis of *p*-hydroxyphenylhydrazone, hydrazine hydrate, and acetylacetone or enaminones under microwave irradiation, thus saving synthesis cost and time. Dyebath reuse in the dyeing of polyester fabrics represents a feasible and economic route to save water and chemical consumption without sacrificing the quality of the finished goods. The dyed polyester fabrics, which display yellowish-orange to dark orange hues, were found to have good fastness to light and excellent fastness to washing and perspiration. Finally, the biological activity of the synthesized dyes against Gram positive bacteria, Gram negative bacteria and yeast were discussed.
